# Substrate Specificity and Peptide Motif Preferences of β-Lytic and L5 Proteases from *Lysobacter* spp. Revealed by LC–MS/MS Analysis

**DOI:** 10.3390/ijms26178603

**Published:** 2025-09-04

**Authors:** Mihail Konstantinov, Leonid Kaluzhskiy, Evgeniy Yablokov, Dmitry Zhdanov, Alexis Ivanov, Ilya Toropygin

**Affiliations:** Institute of Biomedical Chemistry, 10 Building 8, Pogodinskaya Street, 119121 Moscow, Russia; mishanyamihail@ya.ru (M.K.); leonid.kaluzhskiy@ibmc.msk.ru (L.K.); evgeniy.yablokov@ibmc.msk.ru (E.Y.); zhdanovdd@gmail.com (D.Z.); alexei.ivanov@ibmc.msk.ru (A.I.)

**Keywords:** protease specificity, LC–MS/MS, cleavage site analysis, bacterial protease, bioinformatical tools

## Abstract

β-Lytic protease (Blp) and protease L5 are enzymes from *Lysobacter* bacteria with distinct proteolytic and bacteriolytic activities. To characterize their substrate specificity, we employed liquid chromatography–tandem mass spectrometry (LC–MS/MS) analysis following hydrolysis of fractionated protein mixtures. Heatmaps and sequence logos revealed a pronounced specificity of Blp towards glycine and lysine residues, while L5 preferentially cleaved non-polar residues such as methionine, phenylalanine, and leucine. Notably, proline was frequently observed at the P2 position in L5 substrates. Comparative analysis with trypsin revealed that L5 generated significantly shorter peptides, whereas Blp produced fragments similar in length to tryptic peptides. These findings indicate different cleavage preferences and suggest potential applications for these enzymes in proteomic analysis.

## 1. Introduction

Proteases represent one of the most important classes of enzymes, characterised by a broad range of functional activities and diverse applications. They catalyse the hydrolysis of peptide bonds in proteins and are involved in the regulation of numerous biological processes, including digestion, apoptosis, immune responses, and cell differentiation [1,2,3]. Owing to these properties, proteases are widely applied in biotechnology, medicine, and the food and pharmaceutical industries, as well as in textile manufacturing and mining [4,5,6]. Their uses include tissue processing and cheese production; they also serve as diagnostic and therapeutic agents [7,8,9].

As the number of biotechnological and biomedical challenges continues to grow, so does the demand for novel enzymes with improved or unconventional properties. Of particular interest is the search for proteases with unique substrate and molecular specificities, that is, enzymes capable of selectively cleaving specific proteins or retaining activity under non-standard conditions (e.g., high temperatures, extreme pH). Investigating protease specificity is therefore critical for understanding their mechanisms of action, as well as for the discovery and characterisation of natural proteases with valuable functional properties, including those amenable to engineering for specific applications.

Proteases produced by *Lysobacter* bacteria have attracted particular attention. These Gram-negative microorganisms are known for their ability to lyse the cell walls and membranes of other bacteria, making them promising sources of novel antimicrobial agents [10]. The bacteriolytic activity of *Lysobacter* is attributed to the production of various antimicrobial compounds, as well as outer membrane vesicles that can carry these agents and exhibit pronounced proteolytic and lytic properties [11]. Among the proteases secreted by these bacteria, LysC and α-lytic protease (Alp) are well-characterised enzymes that are commercially available and widely used in proteomic research as efficient tools for enzymatic protein digestion [12,13]. However, the functional roles of other secreted *Lysobacter* proteases remain poorly understood, and their substrate specificity has yet to be systematically characterised.

Traditional methods for studying protease specificity rely on the use of synthetic peptide substrates containing chromogenic or fluorogenic labels, followed by spectrophotometric detection [14]. However, in recent years, proteomics-based approaches employing mass spectrometry have gained increasing popularity [15,16,17]. These methods utilise native proteins or even entire proteomes as substrates, thereby eliminating the need for substrate synthesis and greatly expanding the diversity of detectable cleavage sites.

Earlier studies investigated the activity of β-lytic (Blp) proteases from *Sorangium* sp. and *Lysobacter* sp. IB-9374 using a limited set of substrates and demonstrated that these enzymes cleave mainly at Gly–Gly and Gly–Ala [18,19]. These observations provided the first insight into the proteolytic and bacteriolytic properties of Blp proteases but did not allow a comprehensive assessment of their substrate preferences.

**Figure 1:**
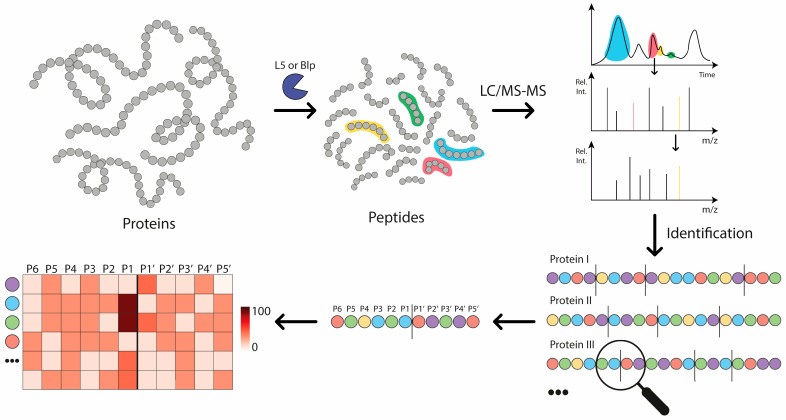
Graphical abstract. General scheme of the experiment to determine the substrate specificity of Blp (*Lysobacter capsici* VKM B-2533^T^) and L5 (*Lysobacter capsici* XL1) proteases. (step 1) A set of proteins was hydrolysed separately by the investigated enzymes. (step 2) The resulting set of peptides was separated using UHPLC coupled with Q-TOF MS, and fragmentation spectra were recorded for the separated peptides. (step 3) These spectra were used to identify the peptides. (step 4) The search results enabled us to determine the frequency of occurrence of hydrolysis sites and their surroundings. (step 5) The obtained data were presented in the form of a heatmap. The *x*-axis shows the hydrolysis sites (P6–P1||P1′–P5), and the *y*-axis shows the amino acids.

Subsequently, we investigated the substrate specificity of Blp from *Lysobacter capsici* VKM B-2533T using matrix-assisted laser desorption/ionisation time-of-flight (MALDI-TOF) mass spectrometry [20], as suitable commercial synthetic substrates are difficult to find, presumably due to the enzyme’s inability to efficiently cleave short peptides. In that study, Blp was shown to preferentially cleave at Gly-X, Ser-X, Lys-X, Ala-X, Val-X, Glu-X, and Phe-X motifs, revealing a broader substrate specificity than previously reported. Nonetheless, this approach has inherent limitations, as it allows only a small number of substrates to be analysed simultaneously and lacks sufficient statistical power to comprehensively identify all preferential cleavage sites. The objective of the present study was to ascertain the molecular specificity of two bacterial proteases isolated from *Lysobacter* outer membrane vesicles, Blp (*Lysobacter capsici* VKM B-2533T) and protease L5 (*Lysobacter capsici* XL1), using LC–MS/MS analysis, which allows the comprehensive profiling of numerous substrates in a single experiment and provides greater statistical robustness compared with the previously applied MALDI-TOF approach (Figure 1). Since substrate preferences may also be influenced by structural motifs affecting backbone flexibility and substrate positioning, the LC–MS/MS approach was chosen as it enables the comprehensive profiling of potential cleavage sites.

## 2. Results

### 2.1. The Analysis of Protease Specificity and the Subsequent Visualisation of the Results

For accurate determination of protease specificity, the origin of the protein substrates is less critical than the diversity of their amino acid sequences. In this study, we combined proteins derived from rat liver and human serum to ensure a broad and representative set of potential cleavage sites. Due to the limited proteome coverage associated with the LC–MS/MS system used, rat liver lysate and human serum samples were separated by SEC (size-exclusion chromatography) and fractionated prior to enzymatic digestion to increase the number of detectable peptides and to improve the overall resolution of the subsequent proteomic analysis.

**Figure 2:**
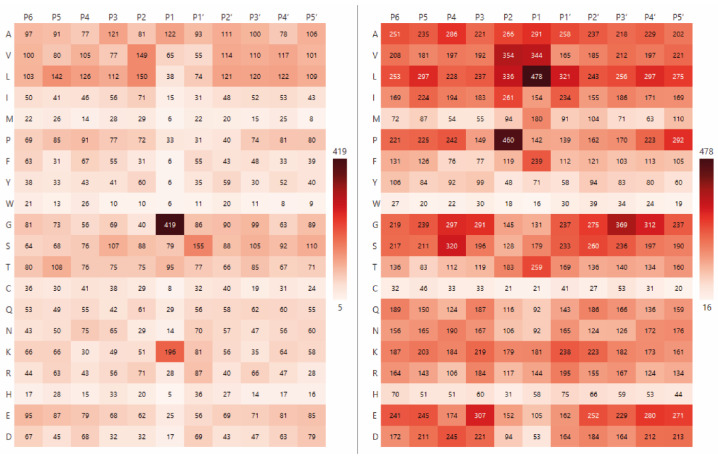
Heatmaps of the raw data frequency of occurrence of the hydrolysis sites of the studied enzymes (Blp on the (**left**), L5 on the (**right**)).

This diverse collection of peptides was then used to analyse the amino acid specificity of the Blp and L5 enzymes. To visualise the positional preferences of each protease, heatmaps were constructed (Figure 2) showing the frequency of cleavage at different amino acid residues at positions P6–P5′. The colour intensity reflects the relative frequency of each residue at a given position, with higher frequencies shown in darker maroon shades.

As demonstrated in Figure 2, Blp exhibits a clear preference for cleaving on glycine residues (G—419 aa, 34.5%) and lysine residues (K—196 aa, 16.1%). With regard to the L5 enzyme, hydrolysis predominantly occurs on non-polar amino acid residues, namely leucine (L—478 aa, 14.8%), valine (V—344 aa, 10.6%), alanine (A—291 aa, 9%) and phenylalanine (F—239 aa, 7.4%), as well as on the polar uncharged amino acid threonine (T—259 aa, 8%). Notably, proline at the P2 position is frequently observed (P—14.3%), which is highlighted in maroon in the right-side plot of Figure 2.

However, amino acids occur at different frequencies, and these frequencies can vary significantly depending on the organism, tissue, or organ. For example, in the entire Swiss-Prot database, tryptophan residues are approximately 8.7 times less frequent than lysine residues, while methionine residues are about 4 times less frequent. This reflects an inherently uneven distribution of amino acids in proteins, which can vary from one organism to another (https://www.uniprot.org/uniprotkb/statistics (accessed on 30 April 2025)). A simple count of cleavage events at a given amino acid position, without considering its overall abundance in the proteome, may lead to conclusions that are not entirely accurate. To obtain a more accurate assessment of protease specificity, we normalised the frequency of cleavage at each amino acid position by dividing it by the background frequency of that residue.

**Figure 3:**
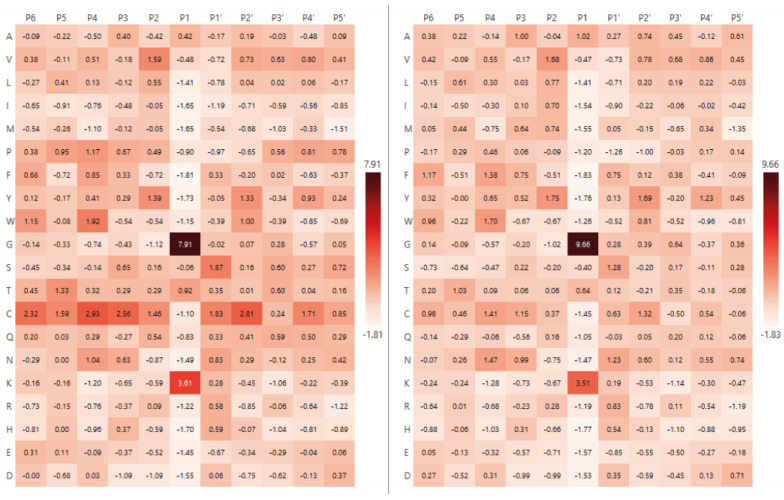
Heatmaps of Blp specificity normalised to Swiss-Prot database composition (**left**) and normalised to experimental dataset amino acid composition (**right**). Data are presented as Z-scores.

**Figure 4:**
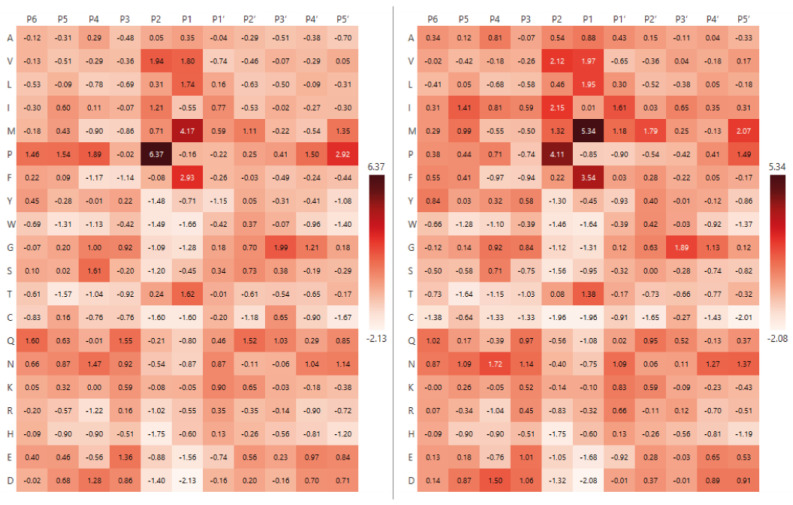
Heatmaps of L5 specificity normalised to Swiss-Prot database composition (**left**) and normalised to experimental dataset amino acid composition (**right**). Data are presented as Z-scores.

To improve interpretability and facilitate visualisation, normalised values were transformed into Z-scores, which represent the deviation of the observed frequency from the expected mean, expressed in standard deviations. This approach allows for a more robust identification of statistically significant preferences and improves the readability of heatmaps. As the background dataset, we used either the global amino acid frequencies from the entire Swiss-Prot database and the amino acid composition of proteins identified in our experiments, respectively. Figure 3 and Figure 4 show the heatmaps for Blp and L5, respectively, following normalisation to the Swiss-Prot amino acid composition and experimental dataset amino acid composition.

As shown in Figure 3 and Figure 4, there were no substantial differences between the amino acid composition normalised to the Swiss-Prot database and the composition derived from proteins identified in our experimental dataset. This observation is further supported by correlation analysis comparing the amino acid composition of the Swiss-Prot database with that of the experimentally identified proteins: Spearman’s and Pearson’s correlation coefficient exceeded 0.9 for each protease. These values indicate that the amino acid composition of the identified proteins closely reflects the overall amino acid distribution observed in the broader Swiss-Prot database. However, normalisation to the amino acid composition of the actual set of proteins subjected to hydrolysis—that is, those residues that were directly accessible to the protease—provides a more accurate representation of enzyme specificity.

While the specificity profile for Blp remained consistent across both normalisation strategies (Gly—Z-score 9.66, 32.5%; Lys—Z-score 3.51, 15.5%), notable changes were observed for L5. As shown in Figure 4, methionine (M—Z-score 5.34, 13.4%), phenylalanine (F—Z-score 3.54, 10.5%), valine (V—Z-score 1.97, 8%) and leucine (L—Z-score 1.95, 8%) were identified as the most frequently occurring residues at the cleavage site. Remarkably, methionine, which had not been among the most abundant residues prior to normalisation, exhibited the highest apparent specificity for this enzyme (Table 1).

**Figure 5:**
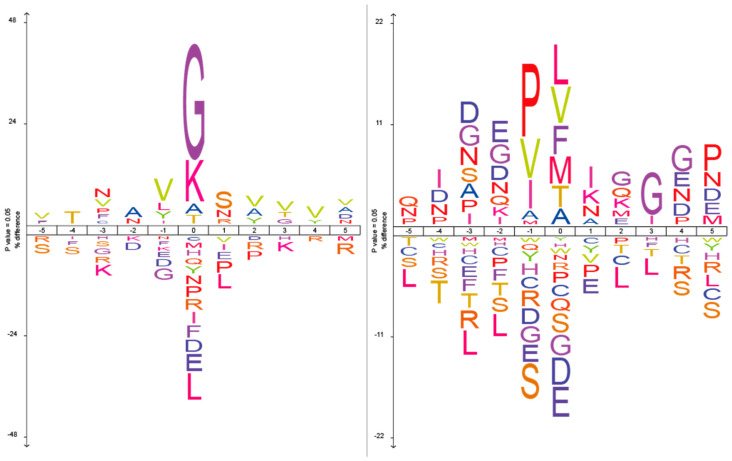
Sequence logos for Blp (**left**) and L5 (**right**). The *X*-axis represents amino acid positions relative to the hydrolysis site (position 0), and the *Y*-axis indicates the percentage difference in amino acid frequency compared to the amino acid distribution from *Rattus norvegicus* proteins in the Swiss-Prot database.

To complement the site-specific analysis, we also generated sequence logos to visualise positional residue frequency patterns relative to the cleavage sites (Figure 5). The percentage difference scale reflects the absolute difference in amino acid frequencies between the experimental dataset and a control background composed of *Rattus norvegicus* proteins. This taxon was selected because the iceLogo web tool does not allow selection of the entire Swiss-Prot database as a background, and the majority of proteins (63%) in our study originated from rat-derived samples.

### 2.2. Analysis of Hydrolysis Motifs for the L5 Protease

A notable feature of L5 protease specificity, as observed in the heatmap (Figure 4), is the prominent occurrence of proline at the P2 position. This residue displays one of the highest Z-scores across the entire matrix, which may indicate a critical role in substrate recognition or binding. Based on this observation, we hypothesised that the enzyme may recognise not only individual amino acid residues, but specific short sequence patterns, such as dipeptide motifs (e.g., P–M, P–F, etc.).

**Figure 6:**
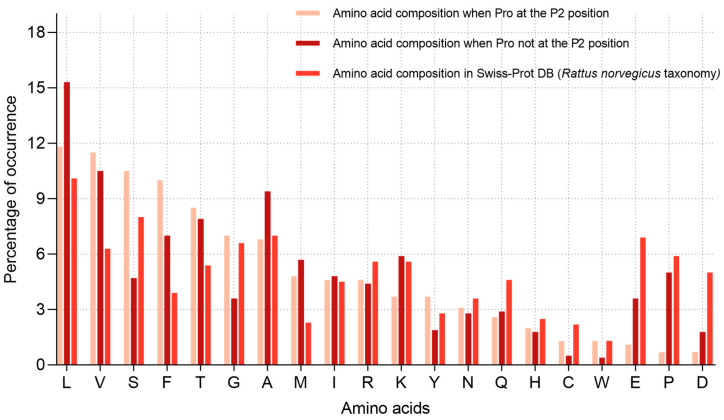
Amino acid distribution in the Swiss-Prot database (*Rattus norvegicus*) and at the P1 position of peptides cleaved by L5 in two contexts: with proline at the P2 position and without proline at P2. Data are shown as percentage frequencies of amino acid occurrence.

To test this hypothesis, we compared the amino acid composition at the P1 position (after the cleavage site) in two groups: (1) peptides with proline at P2, and (2) in all other cases (Figure 6). If L5 exhibited sequence-based specificity (e.g., for motifs of the P–X type), we would expect a more restricted set of residues at P1 in the presence of proline at P2. However, as shown in the diagrams, the amino acid distribution at P1 was largely similar between the two groups. Proline at P2 was accompanied by a comparable range of residues at P1—including L, V, A, S, and T—with only minor variations in their relative frequencies.

**Figure 7:**
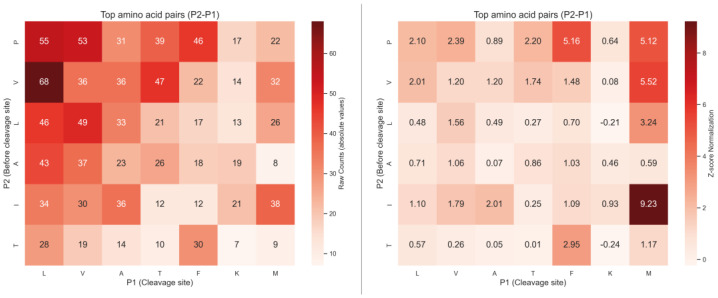
Heatmap of the frequency of P2–P1 amino acid pairs resulting from hydrolysis by the L5 enzyme. The left panel shows the absolute counts of P2–P1 combinations, while the right panel represents values normalised against the background frequencies of amino acid pairs based on the composition of the identified protein dataset.

We also analysed the most frequent pairs of residues at positions P2 and P1. The heatmap in Figure 7 presents the absolute counts of amino acid pairs, with P2 residues on the *Y*-axis (one position upstream of the cleavage site) and P1 residues on the *X*-axis (cleavage site). Despite the overall high frequency of proline at P2, it was observed in combination with a broad variety of P1 residues, including L, F, S, T, and V. To account for amino acid composition biases, we additionally normalised the pair frequencies against the background frequencies of amino acid pairs observed in the set of all identified proteins (Figure 7). After normalisation, the I–M combination showed the highest relative frequency (Z = 9.23), followed by P–F and P–M. However, proline at P2 was still observed together with a wide variety of P1 residues, indicating that the enzyme does not exhibit a strong preference for a single P2–P1 motif but rather acts on multiple sequence contexts.

To further investigate potential sequence motifs near cleavage sites, we performed an analysis of amino acid three-residue patterns (P3–P2–P1). The most frequently observed pattern was G–X–L/M/P (where X is any amino acid), identified in 113 unique peptides.

### 2.3. Comparative Analysis of the Specificity of Blp and L5 Enzymes on the Length of Identified Peptides

A comparative analysis of the lengths of all identified peptides was carried out to assess the specificity of the two proteolytic enzymes, Blp and L5. Only unique peptides were considered in the analysis (which means duplicate sequences were excluded). The average length of peptides generated by Blp-mediated hydrolysis was 14.85 ± 7.26 amino acids (median = 13, mode = 10), whereas peptides produced by L5 were significantly shorter, with an average length of 8.5 ± 2.4 amino acids (median and mode = 8). For comparison with a protease commonly used in proteomics, we analysed peptide lengths obtained from digestion of the same protein mixture with trypsin. In this case, the average peptide length was 13.8 ± 6.7 amino acids (median = 12, mode = 8).

**Figure 8:**
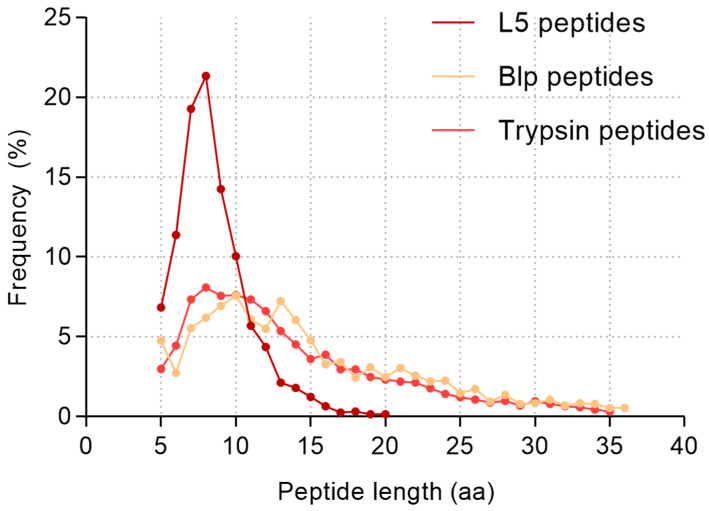
Dependency graph of unique peptide length and frequency of occurrence expressed as a percentage for all enzymes (L5, Blp, and trypsin).

To aid in data interpretation, the frequency distribution of peptide lengths resulting from digestion by each protease is presented as a percentage. Figure 8 illustrates a comparative analysis of the cleavage specificity of Blp and L5 based on the lengths of the identified peptides.

The majority of peptides generated by the L5 protease (over 70% of all identified fragments) were in the range of 5 to 12 amino acids, and no peptides longer than 21 amino acids were observed. In contrast, Blp showed a distribution similar to that of trypsin, generating a considerable proportion of longer peptides, including those exceeding 21 amino acids in length.

**Figure 9:**
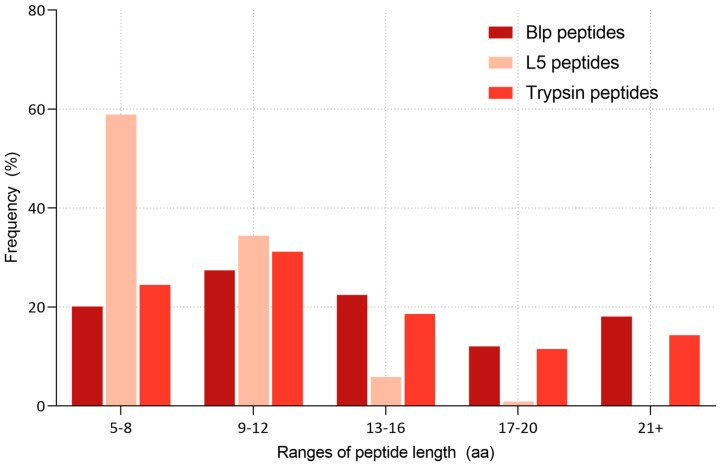
Diagram of the dependence of peptide length ranges on the frequency of occurrence in percent.

Further grouping of peptide lengths into defined ranges (5–8, 9–12, 13–16, 17–20, and >21 amino acids) revealed distinct differences in the hydrolysis patterns (Figure 9).

## 3. Discussion

### 3.1. Enzyme Specificity Analysis

At the initial stage of analysis, using raw (un-normalised) amino acid frequencies at positions P6–P5′, Blp showed a high frequency of glycine (G, 34.5%) and lysine (K, 16.1%) at the P1 position. In contrast, the L5 protease exhibited a broader range of preferences, primarily for non-polar (hydrophobic) residues such as leucine (L—14.8%), valine (V—10.6%), alanine (A—9%), and phenylalanine (F—7.4%), as well as the polar residue threonine (T—8%). After normalisation for amino acid distribution, the specificity profile for Blp remained largely unchanged, confirming its strong preference for glycine (G—32.5%) and, to a lesser extent, lysine (K—15.5%) at the P1 position.

These findings are consistent with previous observations reported by other authors [18,19], where the enzyme’s specificity was assessed using pentaglycine peptides. A similar cleavage profile was also observed in our earlier study employing a set of commercial proteins and MALDI-TOF MS. However, such approaches are limited by the number of accessible substrates, whereas reliable statistical analysis requires at least 30 substrates [21].

**Figure 10:**
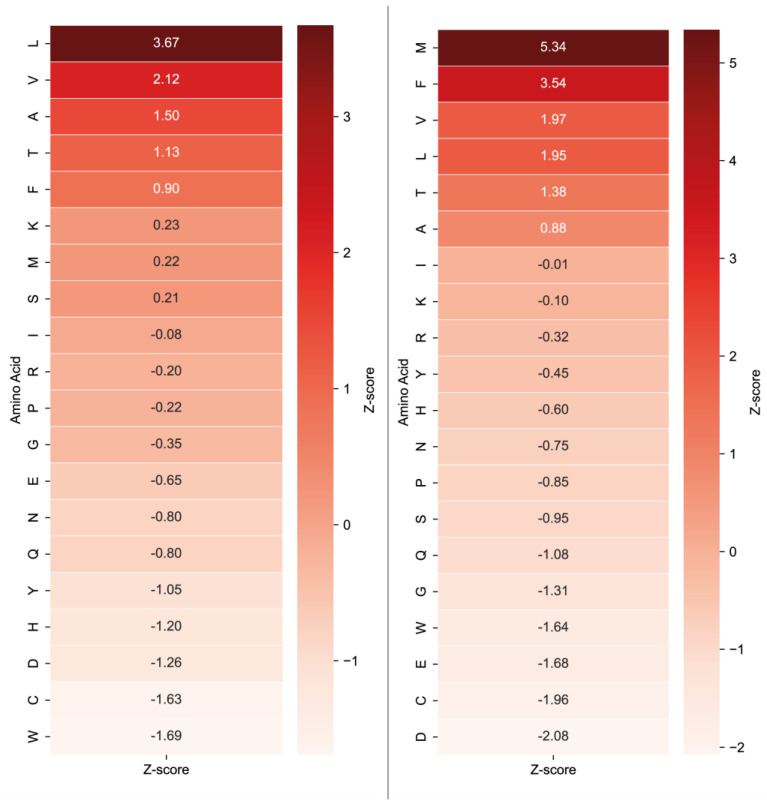
Frequency of occurrence of amino acids at the P1 position in the L5 specificity analysis without (**left**) and with (**right**) consideration of the dataset amino acid composition. In both cases, the values are presented as Z-scores.

In the case of the L5 protease, normalisation substantially altered the substrate specificity preference profile. The enzyme exhibited a pronounced preference for methionine (M—13.4%) and phenylalanine (F—10.5%), while the apparent preference for valine (V—8%) and leucine (L—8%), which were among the most frequent residues before normalisation, was markedly reduced. Notably, all amino acids identified as specific substrates for L5 were non-polar (Figure 10).

One of the most unexpected findings in the analysis of L5 protease specificity was the consistently high likelihood of proline occurring at the P2 position. This residue displayed one of the highest Z-scores across the entire matrix, suggesting a potential role in substrate recognition. In several serine proteases, by contrast, proline is known to inhibit hydrolysis—particularly when located at P1 or P1′—due to its cyclic structure, which restricts backbone flexibility around the peptide bond [22]. However, proline at P2 may play a different, structurally stabilising role, potentially anchoring the peptide in a conformation favourable for catalysis.

To test the hypothesis that proline at P2 may influence amino acid preference at P1, potentially indicating the recognition of a short motif such as Pro–Leu or Pro–Met, we performed a comparative analysis of two sequence groups: peptides with proline at P2 and those without. We also examined the frequency of amino acid pairs (P2–P1) among the identified peptides (Figure 7). While the absolute counts did not reveal a clear dominant Pro–X motif, Z-score normalisation relative to background amino acid pair frequencies showed that certain combinations, such as I–M, P–F, and P–M, occur more frequently than expected. Nevertheless, proline at P2 was still observed in combination with a wide range of P1 residues, suggesting that the enzyme does not exhibit a strong preference for a single P2–P1 motif but, instead, accommodates multiple sequence contexts.

The proteolytic activity of the L5 protease has previously been demonstrated using casein and the synthetic fluorogenic substrate Abz-Ala-Ala-Phe-pNA [23,24]. Specifically, the enzyme was shown to cleave the peptide bond following phenylalanine (Phe–pNA) and to hydrolyse Gly–Gly bonds within the peptidoglycan cross-bridges of *S. aureus*. However, previous analyses of L5 specificity were limited to a narrow range of substrates, preventing comprehensive assessment of the enzyme’s amino acid preferences. Based on our data, we hypothesise that L5 may stabilise its substrates in a defined conformation that facilitates effective binding to the active site. As demonstrated by Vanhoof et al. (1995) [25], proline-containing motifs frequently adopt conformations that are resistant to proteolysis, making cleavage at such sites relatively uncommon. In this context, the high frequency of proline at the P2 position observed for L5 may indicate the enzyme’s ability to recognise or stabilise structurally constrained motifs, thereby overcoming the inherent resistance of proline-containing sites to cleavage. Thus, the prominent occurrence of proline at P2 likely reflects the specific structural preferences of the enzyme.

This hypothesis is consistent with our observations indicating that glycine is rarely present at L5 cleavage sites, suggesting a lack of specificity for this residue. Therefore, the previously reported cleavage of Gly–Gly bonds in the pentaglycine bridge of *S. aureus* peptidoglycan may not reflect an intrinsic specificity of L5 towards glycine, but, rather, the structural accessibility of this region. The flexible and extended conformation of the pentapeptide bridge likely facilitates access to the amide bonds, enabling efficient binding and hydrolysis by the enzyme even in the absence of a strong preference for glycine residues.

Supporting this interpretation is the frequent occurrence of G–X–L/M/P three-residue motifs (where X is any amino acid), observed 113 times among all unique peptides. The prevalence of such three-residue patterns may also reflect a structural preference of L5, for instance, towards flexible regions containing glycine and a hydrophobic residue at the cleavage site. Nevertheless, additional analysis, such as structural modelling, would be required to confirm the relevance of this motif.

It is also possible that L5 specificity is primarily determined by recognition of proline at the P2 position and is not dependent on the identity of the P1 residue. If this were the case, the amino acid distribution at P1 in the presence of proline at P2 would be expected to approximate the natural background distribution (e.g., as represented in the *Rattus norvegicus* composition of the Swiss-Prot database). However, as shown in Figure 6, the amino acid distributions in the two groups differ. Moreover, the total number of cleavage events with proline at P2 (460) is substantially lower than the number of cleavage events without proline in that position (2771), further indicating that proline at P2 is not the sole determinant of L5 specificity.

These observations suggest that L5 specificity may be influenced not only by interactions with individual amino acid residues, but also by the positional or conformational features of the enzyme’s active site. Notably, proline is known to stabilise bends and turns in polypeptide chains [26,27], which could promote a conformation favourable for proper positioning of the substrate in the active site as well as efficient catalysis.

### 3.2. Peptide Length Distribution

Previous studies investigating Blp specificity employed single purified proteins and a set of synthetic substrates bearing fluorescent or chromogenic labels [18,19,28]. As noted above, the use of a single protein is insufficient for a comprehensive assessment of protease specificity. The same limitation applies to synthetic substrates, which typically feature a restricted amino acid repertoire. Moreover, in our earlier work, we were unable to hydrolyse short synthetic peptides containing glycine residues. This may be related to our current observation that Blp is capable of missing potential cleavage sites.

We examined the frequency of missed cleavages in peptides generated by Blp digestion. Among the 629 unique peptides identified, 44% contained one or more missed cleavages when considering glycine (G) as the only cleavage residue, or 33.5% when considering both glycine and lysine (G + K). This suggests that, although glycine-specific sites are present, the enzyme does not cleave at all accessible positions. Examples of peptides with missed cleavage site—where corresponding cleavage fragments were not detected despite having masses suitable for identification—are provided in Appendix A (Table A1).

To further investigate enzyme preferences regarding product size, we analysed peptide length distributions for both proteases. Blp produced peptides with an average length of 14.85 ± 7.26 amino acids, whereas the average peptide length for L5 was only 8.5 ± 2.4 amino acids. A comparative analysis of the grouped peptide lengths (5–8, 9–12, 13–16, 17–20, and >21 amino acids) is shown in Figure 9.

As a reference, we included data from digestion of the same human serum protein fractions used in the Blp experiments, performed with the “classical” protease trypsin. This difference in the length of cleavage products may reflect a less extensive or more selective hydrolytic mechanism. The tendency of L5 to generate shorter peptides might be related to differences in the steric organisation of its active-site subsites compared with Blp. Previous studies have shown that variations in the S2 and S3 pockets of proteases can significantly influence substrate preferences and even alter the ability to cleave substrates with specific residues, such as Pro, in P2 [29].

Although L5 exhibits broad amino acid specificity and tends to generate short fragments, previous studies have shown that its bacteriolytic activity is substantially lower in the soluble form compared to when it is secreted via outer membrane vesicles [30]. This suggests that L5 may not function as a primary lytic enzyme targeting cell wall degradation, but, rather, as a supporting protease within the bacterial secretome. In contrast, Blp displays higher bacteriolytic activity and a narrower specificity—primarily cleaving after glycine residues—which may indicate targeted degradation of specific cell wall components. Therefore, L5 and Blp may fulfil complementary roles: Blp acting as the primary lytic agent, while L5 serves as a more adaptive enzyme with broader but less aggressive specificity.

The tendency of Blp to generate longer peptides may reflect a more selective cleavage pattern. A similar digestion profile, characterised by the formation of a limited number of longer peptides, has also been observed for Alp [13]. Interestingly, WaLP (wild-type Alp) and MaLP (mutant-type Alp), cleave predominantly after aliphatic amino acid residues (WaLP: A, V, T, and S; MaLP: L, F, and V), which is comparable to L5 in terms of residue preference. However, the average peptide lengths generated by WaLP (11.7 ± 4.8 amino acids) and MaLP (12.0 ± 4.5 amino acids) are more similar to Blp than to L5. The shorter peptides produced by L5 may result from sequential, multiple cleavage events—a feature often associated with enzymes involved in bacteriolytic processes.

## 4. Materials and Methods

### 4.1. Samples and Chemicals

Tris(2-carboxyethyl)phosphine (TCEP) (Thermo Scientific, Waltham, MA, USA, T2556), 2-chloroacetamide (CAA) (Sigma, St. Louis, MO, USA, C0267), formic acid (FA) (Merck Millipore, Burlington, MA, USA, 5.43804.0100), LC–MS grade water (neoFroxx GmbH, Einhausen, Germany, LC-10237.2), LC–MS grade acetonitrile (ACN) (Biosolve Chimie, Dieuze, France, 012078), ammonium bicarbonate buffer (pH 7.8) (Sigma-Aldrich, St. Louis, MA, USA, 1066-33-7), phosphate buffer saline (PBS) (137 мM NaCl, 2.7 мM KCl, 10 мM Na_2_HPO_4_, 1.8 мM KH_2_PO_4_, pH 7.4), protease inhibitor mix (GE Healthcare, Chicago, IL, USA, #80-6501-23), SEC buffer (10 мM HEPES (pH 7.4), 150 мM NaCl, 3 мM EDTA, 1 мM DTT, 0.5% NaN_3_ и 0.05% Tween 20).

Blp and L5 were expressed and purified as previously described [23,30,31].

### 4.2. Preparation of Intact Liver Tissue Lysate

Male Wistar rats (*Rattus norvegicus*), 5 months old, were used to obtain tissue samples. The animals were kept under natural light and had free access to standard food and water. The initial weight of the animals ranged from 200 to 240 g, and they were euthanised via decapitation under ether anaesthesia (AVMA Guidelines for the Euthanasia of Animals: 2020 Edition). Liver samples were collected, washed with 0.9% NaCl, and subsequently frozen in liquid nitrogen. The experiment involved three rats.

Approximately equal masses of liver tissue fragments obtained from each of the three rats were pooled into a mixed sample to minimise individual variation. The tissue fragments were then ground in a glass mortar and pestle in a phosphate buffer saline, PBS (137 mM NaCl, 2.7 mM KCl, 10 mM Na_2_HPO_4_, 1.8 mM KH_2_PO_4_, pH 7.4), containing a protease inhibitor mix. Subsequently, the lysate was resuspended with a Silent Crusher S rotary homogeniser (Heidolph, Schwabach, Germany).

### 4.3. Human Blood Serum Sample Collection

A 1 mL sample of human blood serum was obtained from a single healthy donor (female, 30 years old) (supplier: ABM LLC, St. Petersburg, Russia).

### 4.4. Adherence to Ethical Standards

The experiments were conducted in accordance with the generally accepted norms of the humane treatment of laboratory animals and in compliance with the Order of the Ministry of Health of the Russian Federation No. 199n (1 April 2016), entitled “On Approval of the Rules of Good Laboratory Practice,” and Directive 2010/63/EU of the European Parliament and Council of the European Union on the protection of animals used for scientific purposes (22 September 2010).

### 4.5. Size-Exclusion Chromatography (SEC) Fractionationating

Gel chromatographic fractionation of protein material from rat liver tissue lysate by molecular weight was performed on an AKTA Purifier 10 chromatograph (Cytiva, Marlborough, MA, USA) under the control of the UNICORN v5.31 program. A 2 mL sample of lysate (~40 mg total protein) was incubated for 1 h at +4 °C to establish the equilibrium of the protein complexes. The lysate samples were then centrifuged at 12,000× *g* for 10 min at 4 °C to remove any precipitate. The lysate was then fractionated on a HiLoad 16/600 column with Superdex 200 prep grade (Cytiva, USA), pre-equilibrated with SEC buffer, at a flow rate of 800 μL/min and a temperature of 15 °C. Six SEC fractions were collected for further mass spectrometric protein identification. The concentration of total protein in the collected fractions was determined using a calibration curve at 280 nm on a QE65000 spectrophotometer with a DH-2000 light source (Ocean Optics, Orlando, FL, USA); the measured concentrations of total protein ranged from 0.2 to 1 mg/mL.

Serum fractionation was also performed using an AKTA Purifier 10 system (GE Healthcare Life Sciences, Chicago, IL, USA) equipped with a Superose 6 10/300 GL gel filtration column (Cytiva, USA). A 100 µL sample of human serum was injected. The chromatographic system operated at a constant flow rate of 0.3 mL/min throughout the experiment. The temperature of the chromatography chamber was maintained at 4 °C, and protein elution was monitored by absorbance at 280 nm. Fraction collection was initiated after 4 mL of eluent had passed following sample injection; a total of 10 fractions were collected in 500 µL volumes.

The total protein concentration for both serum and liver lysate fractions was determined by the Bradford assay using a ClarioStar plate reader (BMG, Berlin, Germany).

### 4.6. Hydrolysis of Proteins

Reduction and alkylation of cysteine of protein in all fractions was performed by adding 10 mM TCEP solution and 40 mM CAA to each fraction containing 10 μg of total protein, followed by incubation for 15 min at 37 °C. Proteolysis was carried out by adding 3 μL (46 μg/mL) of L5 enzyme and 50 мM of ammonium bicarbonate buffer to each fraction. Hydrolysis with Blp enzyme was performed under the same conditions, but the enzyme concentration was 289 μg/mL. Digestion with trypsin was carried out under the same protocol, using the enzyme, at a final concentration of 20 μg/mL. Samples were incubated overnight (about 16 h) at 37 °C in thermostat BE 400 (MEMMERT, Büchenbach, Germany).

### 4.7. LC–MS/MS Analysis

LC–MS/MS analysis was performed on a micrOTOF-QII mass spectrometer (Bruker Daltonik, Bremen, Germany) equipped with a CaptiveSpray ion source, coupled to a nanoElute UHPLC system (Bruker Daltonik, Bremen, Germany). Prior to analysis, samples were desalted using C18 StageTips (Supelco, Merck, USA), following the protocol described by Rappsilber et al. [32]. Dried samples were reconstituted in 0.1% FA and 1–6 μL of each sample was injected onto an Acclaim™ PepMap™ C18 trap column (5 μm, 0.3 mm × 5 mm; Thermo Fisher Scientific, Waltham, MA, USA) under 400 bar pressure. The injection volume varied depending on peptide yield after desalting and drying using a SpeedVac concentrator (Eppendorf, Hamburg, Germany).

Peptides were separated on an Aurora Ultimate CSI C18 column (1.7 μm, 120 Å, 75 μm × 250 mm; IonOpticks, Collingwood, Australia) at 50 °C and a flow rate of 300 nL/min, using a linear gradient from 2% to 85% solvent B (0.1% FA in ACN) against solvent A (0.1% FA in water) with a total chromatographic time of 100 min. The gradient profile was 2–3% B (0–1 min), 3–17% B (1–57 min), 17–25% B (57–78 min), 25–34% B (78–92 min), 34–85% B (92–93 min); it was held at 85% B for 7 min, then re-equilibrated to 2% B.

Mass spectrometry was carried out in positive ion mode with a capillary voltage of 1500 V, dry gas temperature of 150 °C, and gas flow of 3 L/min. MS1 scans were acquired over the m/z range 150–2200 at 2 Hz. MS/MS spectra were recorded in automated mode using dynamic precursor selection (charge states 2+–4+) with a cycle time of 3 s and spectral acquisition rates of 8–32 Hz. Peptide fragmentation was achieved using collision-induced dissociation (CID) in data-dependent acquisition mode (DDA). The collision energy was automatically optimised based on precursor m/z and charge state.

Raw data were processed using Compass DataAnalysis 5.1 software (Bruker Daltonik, Bremen, Germany). Peak lists were exported in Mascot Generic Format (*.mgf, available in Supplementary Materials) and searched against the Swiss-Prot database using Mascot Server version 2.3.0 (Matrix Science, London, UK). Searches were performed with the following parameters: enzyme specificity—none; variable modifications—deamidation (NQ), oxidation (M), and carbamidomethylation (C); peptide mass tolerance—4.5 ppm; fragment mass tolerance—0.2 Da; allowed precursor charge states—2+, 3+, 4+. Species-specific taxonomies were selected: *Rattus norvegicus* for rat liver lysate samples and *Homo sapiens* for serum samples. For Blp specificity analysis, 8 hydrolysis samples were analysed, resulting in the identification of 314 proteins. L5 enzyme specificity analysis included 9 samples, with 520 proteins identified.

### 4.8. Bioinformatic Analysis

Validation of identifications was performed using two approaches. The standard method in proteomics relies on calculating the false discovery rate (FDR) from target and decoy database matches, with a threshold of ≤1% commonly used. However, accurate FDR estimation requires a sufficient number of identifications (typically >100 per sample). In our case, several samples yielded fewer than 100 protein hits, primarily due to the limited proteolytic efficiency of the studied enzymes and instrument sensitivity. Under these conditions, FDR values become unstable and prone to overestimation (e.g., a single decoy hit among 50 proteins yields 2% FDR) [33].

To overcome this limitation, we applied an alternative confidence filter based on the Mascot Ion Score—a parameter reflecting the quality of spectral matching. Ion Score thresholds ranging from 1 to 25 were tested, and specificity profiles were evaluated at each level using heatmaps. At thresholds below 20, profiles were inconsistent and likely influenced by random matches. From Ion Score ≥ 20, specificity patterns stabilised and remained unchanged with further increases. Therefore, a cut-off of 20 was chosen for all samples to maximise data confidence while minimising false positives.

Acquired data were exported with mostly default parameters except the following: export format—XML, significance threshold set to *p* < 0.05 with the inclusion of protein sequences in the hit output. For the Ion Score threshold (cut-off parameter), we chose 20.

All identification results were processed using custom-built scripts. Mascot search result files in XML format were parsed to extract all confidently identified peptides. To determine cleavage sites, the amino acid residue before the peptide and the last residue of the peptide were extracted, excluding N-terminal methionine (if present) and the final residue in the C-terminal peptide, where applicable. Each unique cleavage site position was considered only once, and duplicate entries were excluded. For each confidently assigned cleavage site, a fixed-length sequence window comprising five amino acids upstream and five downstream of the scissile bonds was extracted from the parent protein sequence. The resulting output was formatted as a position-specific scoring matrix (PSSM), where amino acid positions were mapped according to standard protease cleavage site nomenclature: P6, P5, P4, P3, P2, P1 || P1′, P2′, P3′, P4′, P5′—with P1 denoting the cleavage site. Heatmaps were generated from the obtained data using homemade visualisation scripts. In addition, sequence logos were constructed using the IceLogo web tool [34]. The scoring system was set as percentage (percent difference in the frequency of amino acids at a location); *Rattus norvegicus* were selected as the reference set, *p* value 0.05.

Correlation between the amino acid composition of the Swiss-Prot database and the experimentally identified proteins was assessed using both Pearson’s and Spearman’s correlation coefficients. The calculations were performed in Microsoft Excel (Microsoft, Redmond, WA, USA).

To assess repetitive motifs during hydrolysis by L5 protease, amino acids located at positions P3, P2, and P1—that is, three positions before the cleavage site, two positions before, and immediately before—were extracted from each unique peptide identified by LC–MS/MS analysis (taking into account the cleavage site annotation). A Python script (version 3.9) was used for this purpose. Peptides that were duplicated in sequence were counted only once to avoid bias in the statistics due to repeated identifications. Combinations of residues at positions P2 and P1 were used to calculate the most frequent amino acid pairs. The resulting frequencies of occurrence of pairs (P2-P1) were visualised as a heatmap. Three-residue patterns in the format P3–P2–P1 were also analysed. All extracted three-residue patterns were grouped and sorted by frequency of occurrence. The results of the analyses were used to discuss possible structural or sequential specificity.

Scripts availability in Supplementary Materials.

## 5. Conclusions

This study provides a comprehensive analysis of the substrate specificity of two bacterial proteases, Blp and L5, based on LC–MS/MS data and visualisation of cleavage site preferences. Using proteins from rat liver and human serum ensured a broad range of accessible cleavage sites.

L5 generated predominantly short peptides and displayed frequent cleavage near proline residues, particularly at the P2 position, suggesting a possible structural preference rather than “classical” residue-based selectivity. In contrast, Blp exhibited narrower specificity with a strong tendency to cleave after glycine, often producing longer peptides. The observed cleavage patterns, visualised through heatmaps and sequence logos, highlight the potential mechanistic differences between these enzymes. Blp seems to act as a more selective bacteriolytic enzyme, whereas L5 functions as a broadly active protease capable of processing structurally constrained motifs.

Our findings expand the current understanding of the specificity of bacterial proteases and provide a foundation for future studies into their biological roles and potential applications in biotechnology and antimicrobial development.

## Figures and Tables

**Table 1:** Relative frequencies (%) of the most frequently observed amino acids at the cleavage sites before and after normalisation.

Amino Acid	Blp (%)	Blp Norm (%)	L5 (%)	L5 Norm (%)	Group
G	34.5	32.5	–	–	Non-polar
K	16.1	15.5	–	–	Polar charged
L	–	–	14.8	8	Non-polar
V	–	–	10.6	8	Non-polar
A	–	–	9	6.3	Non-polar
F	–	–	7.4	10.5	Non-polar
T	–	–	8	7.1	Polar uncharged
M	–	–	5.5	13.4	Non-polar

## Data Availability

Data available in Supplementary Materials.

## Supplementary Materials

The following supporting information can be downloaded at https://www.mdpi.com/article/10.3390/ijms26178603/s1.

## Abbreviations

The following abbreviations are used in this manuscript:

Alp	α-lytic protease
Blp	β-lytic protease
WaLP	wild type α-lytic proteas
MaLP	mutant type α-lytic protease
SEC	size-exclusion chromatography
LC–MS/MS	liquid chromatography–tandem mass spectrometry
MALDI-TOF MS	matrix-assisted laser desorption/ionisation time-of-flight mass spectrometry
UHPLC	ultra-high-performance liquid chromatography
ACN	acetonitrile
FA	formic acid

## Appendix A

**Table A1:** Identified peptides with missed cleavage site.

Protein	Peptide with Missed Cleavages	Missed Cleavages	Expected Unidentified Fragments
CPSM_RAT	HPSSVA**G**EVV	1	HPSSVAG, EVV
CPSM_RAT	HPSSVA**G**EVVFNT	1	HPSSVAG, EVVFNT
CPSM_RAT	EVI**K**AERPD**G**LILG	2	EVIK, AERPDG, LILG, EVIKAERPDG, AERPDGLILG
SVEP1_MOUSE	SDP**K**IQLIFNIT	1	SDPK, IQLIFNIT
CPSM_RAT	ADTI**G**YPVMIRS	1	ADTIG, YPVMIRS
CPSM_RAT	NMENVDAM**G**VHTG	1	NMENVDAMG, VHTG
CPSM_RAT	QSVDFVDPN**K**QNLIAEVSTK	1	QSVDFVDPNK, QNLIAEVSTK
ALDH2_MESAU	STEV**G**HLIQVA	1	STEVG, HLIQVA
ALDH2_MESAU	SHEDVD**K**VAFTG	1	SHEDVDK, VAFTG
ALDH2_MESAU	NPFDSRTEQ**G**PQVDETQFK	1	NPFDSRTEQG, PQVDETQFK
BHMT1_RAT	RQVADE**G**DALVAG	1	RQVADEG, DALVAG
JHD2C_MOUSE	NSQAV**G**ERRL	1	NSQAVG, ERRL
MVP_RAT	AARIE**G**E**G**SVLQA	2	AARIEG, EG, SVLQA…
